# Parabiosis Incompletely Reverses Aging-Induced Metabolic Changes and Oxidant Stress in Mouse Red Blood Cells

**DOI:** 10.3390/nu11061337

**Published:** 2019-06-14

**Authors:** Evan J. Morrison, Devin P. Champagne, Monika Dzieciatkowska, Travis Nemkov, James C. Zimring, Kirk C. Hansen, Fangxia Guan, Derek M. Huffman, Laura Santambrogio, Angelo D’Alessandro

**Affiliations:** 1 Department of Biochemistry and Molecular Genetics, University of Colorado Denver – Anschutz Medical Campus, 12801 East 17th Ave RC1 South, Aurora, CO 80045, USA; evan.morrison@ucdenver.edu (E.J.M.); devin.champagne@ucdenver.edu (D.P.C.); monika.dzieciatkowska@ucdenver.edu (M.D.); travis.nemkov@ucdenver.edu (T.N.); kirk.hansen@ucdenver.edu (K.C.H.); 2 BloodWorks Northwest, Seattle, WA 98104, USA; JZimring@BloodWorksNW.org; 3 Departments of Medicine, Albert Einstein College of Medicine, 1300 Morris Park Avenue, New York, NY 10461, USA; fangxia.guan@einstein.yu.edu (F.G.); derek.huffman@einstein.yu.edu (D.M.H.); 4 Institute for Aging Research, Albert Einstein College of Medicine, 1300 Morris Park Avenue, New York, NY 10461, USA; 5 Department of Molecular Pharmacology, Albert Einstein College of Medicine, 1300 Morris Park Avenue, New York, NY 10461, USA; 6 Department of Pathology, Microbiology & Immunology, Albert Einstein College of Medicine, 1300 Morris Park Avenue, New York, NY 10461, USA; laura.santambrogio@einstein.yu.edu; 7 Department of Medicine—Division of Hematology, University of Colorado Denver—Anschutz Medical Campus, 12469 East 17th Ave RC2, Aurora, CO 80045, USA

**Keywords:** erythrocyte, metabolism, mass spectrometry, blood, metabolomics

## Abstract

Mature red blood cells (RBCs) not only account for ~83% of the total host cells in the human body, but they are also exposed to all body tissues during their circulation in the bloodstream. In addition, RBCs are devoid of de novo protein synthesis capacity and, as such, they represent a perfect model to investigate system-wide alterations of cellular metabolism in the context of aging and age-related oxidant stress without the confounding factor of gene expression. In the present study, we employed ultra-high-pressure liquid chromatography coupled with mass spectrometry (UHPLC–MS)-based metabolomics and proteomics to investigate RBC metabolism across age in male mice (6, 15, and 25 months old). We report that RBCs from aging mice face a progressive decline in the capacity to cope with oxidant stress through the glutathione/NADPH-dependent antioxidant systems. Oxidant stress to tryptophan and purines was accompanied by declines in late glycolysis and methyl-group donors, a potential compensatory mechanism to repair oxidatively damaged proteins. Moreover, heterochronic parabiosis experiments demonstrated that the young environment only partially rescued the alterations in one-carbon metabolism in old mice, although it had minimal to no impact on glutathione homeostasis, the pentose phosphate pathway, and oxidation of purines and tryptophan, which were instead aggravated in old heterochronic parabionts.

## 1. Introduction

Over the last 160 years, the average life expectancy rose from 45 to ~85 in most industrialized countries [1]. With an increase in longevity worldwide, age-associated non-communicable diseases emerged as a substantial burden in disease incidence and healthcare costs [2]. The list of diseases for which age is an independent risk factor includes current leading causes of mortality in the adult population, such as cancer, diabetes, cardiovascular disease, and neurodegenerative diseases [3]. Despite decades of studies on the topic, the identification of strategies to slow the aging process is frustrated by the complex and stochastic nature of progressive biological decay. Indeed, aging is a complex phenomenon, one that is impacted by several factors such as genetics and environment, and their interplay [4]. Variation in lifespan among species or individuals is indeed dictated in part by genetics [5], although environmental factors such as diet [6], exercise [7], or other lifestyle habits (e.g., smoking [8]) are increasingly appreciated as key contributors to aging. All these factors were shown to contribute to mitochondrial dysfunction and systemic oxidant stress [9,10], etiological contributors to many of the cardiovascular, neurocognitive, and inflammatory complications associated with aging. Meanwhile, interventions that succeeded in slowing features of aging, such as dietary restriction (DR) or rapamycin, are able to potently modulate key metabolic pathways, including insulin/Insulin-like growth factor 1 (IGF-1) signaling, AMP-activated protein kinase (AMPK)/mechanistic target of rapamycin (mTOR), and inflammation [3]. Indeed, several recent studies—from animal models to clinical trials [11]—showed that dietary interventions aimed at reprogramming system metabolism could represent a viable strategy to extend the lifespan, or at least to extend the population health span.

Owing to their capillary distribution, red blood cells (RBCs)—the most abundant human cell in our body (~83% of total human cells excluding bacteria [12])—represent a perfect model to investigate system metabolism. While originally considered simple bags of hemoglobin, RBCs are increasingly being appreciated owing to their complex capacity to buffer system metabolism, as a result of their ~3000 proteins and ~100 molecular membrane transporters [13]. During their 120-day lifespan in the bloodstream, at full oxygen saturation of the ~250–270 million copies of hemoglobin/cell [14], RBCs could transport over one billion/molecules of oxygen per cell—numbers that make it easy to appreciate the likelihood of radical-generating reactions in a cell that is also loaded with >60% of bodily iron [15]. In addition, owing to the lack of nuclei and organelles, mature RBCs cannot synthesize new proteins to face sudden or progressive increases in the oxidant stress challenge. As such, RBCs are a perfect model to investigate system metabolism in the context of cellular oxidant stress without the confounding contribution of de novo synthesis of gene products. Interestingly, the very process of increased inflammation and oxidant stress in aged individuals (also referred to as “inflammaging” [16]) is associated with a stressed erythropoiesis phenotype, one that ultimately results in a skewed hematopoietic maturation toward myeloid progenitors [17]—a potential etiological contributor to the so-called “anemia in the elderly” [18].

RBCs are well equipped with antioxidant systems, especially those depending on glutathione. However, studies from the late 1970s identified a progressive decline in the glutathione-dependent RBC antioxidant capacity as a function of organism and RBC age in mice [19]. This decline is in part explained by the progressive deregulation of the pentose phosphate pathway (PPP) in the aging RBCs of the aging organism (mouse or human [20,21,22]). Indeed, the PPP generates the reducing co-factor Nicotinamide adenine dinucleotide phosphate (NADPH), which is critical for fueling the recycling of several antioxidant systems in RBCs such as glutathione peroxidase, catalase, peroxiredoxins [23], glutaredoxins, thioredoxin reductase system, biliverdin reductase B [24], and the ascorbate–tocopherol axis [25]. Glucose 6-phosphate dehydrogenase (G6PD) is the rate-limiting enzyme of the PPP and, thus, a critical enzyme in NADPH homeostasis in RBCs and other tissues [26]. Nonetheless, ~400 million people suffer from mutations in G6PD that impact its activity to a variable extent, a condition referred to as G6PD deficiency—the most common enzymopathy in humans [27]. Of note, G6PD activity was suggested to decline with age [20,21,22], while transgenic mice overexpressing G6PD have improved health spans [28]. However, despite these classic studies, no recent reports addressed this aspect of RBC biology in the context of aging with state-of-the-art omics approaches. 

In the last few years, the advent of omics technologies revived interest around blood metabolism as a critical source of information to investigate derangements in system metabolism as a function of aging [29]. Recently, we reported the impact of aging on RBC metabolism in a cohort of 97 subjects, including individuals with Down syndrome, the most common human condition due to aneuploidy in the human population (one in ~700 live births in the United States) [30]. Of note, individuals with Down syndrome are more susceptible to several of the comorbidities associated with aging, including neurocognitive diseases (e.g., Alzheimer’s disease), several autoimmune and hematological cancers, pulmonary hypertension, and hearing and vision problems [31,32]. Interestingly, RBC lifespan is shortened in individuals with Down syndrome, a phenomenon that is accompanied by metabolic alterations in one-carbon metabolism (and associated damage-repair mechanisms [33]), and glutathione and purine oxidation, as well as tryptophan metabolism [30]—a pathway critical to immunoregulation [34]. Recently, tryptophan catabolites via inflammatory (interferon signaling) and oxidative metabolism were associated with neurocognitive impairment in Down syndrome [35]. 

Parabiosis, which is a surgical approach for joining the circulatory systems between young–young and old–old (isochronic) or young–old (heterochronic) animals, re-emerged over the past decade in aging research. Studies based on this approach demonstrated that aging occurs as a complex interaction of cell autonomous and cell non-autonomous mechanisms. Using heterochronic parabiosis, cell non-autonomous effects were demonstrated by transposition of aging phenotypes (i.e., old to young, young to old) in several organs and cells, and implicated specific gerontic factors, including beta-2 microglobulin (B2M), C-C Motif Chemokine Ligand 11 (CCL11), and transforming growth factor beta (TGFβ), in mediating these effects. Of note, some of the molecular and behavioral signatures associated with neurocognitive decline in aging were restored in old mice exposed to young blood via heterochronic parabiosis [36]. Recent work showed that the young environment ameliorates neurocognitive defects in aging mice, through mechanisms at least in part involving Tet2 and DNA methylation status in the aged hippocampus [37]. Therefore, in the present study, we employed state-of-the-art metabolomics and proteomics technologies to investigate RBC metabolism in mice of six, 15, and 25 months of age, and to further determine the extent, if any, to which shifts in the metabolome or proteome of the cells are modulated by the systemic environment through heterochronic parabiosis. 

## 2. Materials and Methods

### 2.1. Aging and Parabiotic Mice 

Parabiosis surgery was carried out by the Einstein Chronobiosis and Energetics/Metabolism of Aging Core in young and old male C57BL/6 mice obtained from the National Institute of Aging aged rodent colony at four or 18 months of age, respectively, as described previously [38,39]. Surgical unions were performed between young (four months old) animals (isochronic; young (Y)–Y; *n* = 4), old (18 months old) animals (isochronic old (O)–O; *n* = 5), and young and old mice (heterochronic Y–O; *n* = 5). Following surgery, animals were kept on a partial heating pad overnight. Pairs were then intensively monitored and received subcutaneous (SQ) injections of Banamine (2 mg/kg each) immediately post-op and bis in die (b.i.d.) for three days and then once daily for four days. Animals also received 1 mL of ringer’s lactate SQ immediately after, daily for three days post-op to prevent dehydration. Animals remained joined for ~8 weeks prior to sacrifice. All experimental procedures were approved by the Institutional Animal Care and Use Committee (IACUC) at the Albert Einstein College of Medicine.

### 2.2. Metabolomics 

#### 2.2.1. Sample Extraction 

Metabolomics analyses were performed on 20 μL of packed RBCs at 1:25 dilution in separate extractions of with either methanol:acetonitrile:water (5:3:2 *v/v*) or pure methanol (Optima, Thermo Fisher) prior to vortexing for 30 min at 4 °C and centrifugation at 18,000× *g* for 10 min at 4 °C, as described previously [40,41]. Extracts were analyzed via ultra-high-pressure liquid chromatography coupled with mass spectrometry (UHPLC–MS). 

#### 2.2.2. UHPLC–MS Analysis 

The analytical platform employs a Vanquish UHPLC system (Thermo Fisher Scientific, San Jose, CA, USA) coupled online to a Q Exactive mass spectrometer (Thermo Fisher Scientific, San Jose, CA, USA), as extensively described in prior work [40,42,43,44]. Metabolites were separated with a combination of isochratic and gradient-based methods as per protocols extensively detailed in recent methods papers [45,46]. Briefly, the analytical platform employs a Vanquish UHPLC system coupled online to a Q Exactive mass spectrometer (Thermo Fisher Scientific, San Jose, CA, USA). Samples were resolved over a Kinetex C18 column, 2.1 × 150 mm, 1.7 µm particle size (Phenomenex, Torrance, CA, USA) equipped with a guard column (SecurityGuard™ Ultracartridge UHPLC C18 for 2.1 mm inner diameter (ID) Columns; AJO-8782; Phenomenex, Torrance, CA, USA) using an aqueous phase (A) of water and 0.1% formic acid and a mobile phase (B) of acetonitrile and 0.1% formic acid for positive ion mode runs, while, for negative ion mode runs, 2 mM ammonium acetate was used to replace formic acid. Samples were eluted from the column using either an isocratic elution of 5% B flowed at 250 µL/min and 25 °C or a gradient from 0–5% B over 0.5 min, 5–95% B over 0.6 min, held at 95% B for 1.65 min, 95–5% B over 0.25 min, and held at 5% B for 2 min, flowed at 450 µL/min and 35 °C. The Q Exactive mass spectrometer (Thermo Fisher Scientific, San Jose, CA, USA) was operated independently in positive or negative ion mode, scanning in full MS mode (2 μscans) from 60 to 900 *m*/*z* at 70,000 resolution, with 4 kV spray voltage, 45 sheath gas, and 15 auxiliary gas. Calibration was performed prior to analysis using the Pierce™ Positive and Negative Ion Calibration Solutions (Thermo Fisher Scientific, Waltham, MA, USA). Acquired data was then converted from raw to mzXML file format using Mass Matrix (Cleveland, OH, USA). Samples were analyzed in randomized order with a technical mixture injected after every 15 samples to qualify instrument performance. Metabolite assignments were performed using MAVEN (Princeton, NJ, USA), [47] against an in-house library of stable isotope-labeled standards [41].

#### 2.2.3. Proteomics Analyses 

Mouse RBC proteomics analyses were performed via filter-aided sample preparation (FASP), followed by trypsinization and nanoUHPLC–MS/MS analyses (nanoEasy LC II coupeld to QExactive HF, Thermo Fisher), as extensively described in prior work [13]. In the interest of space, the interested reader is referred to previous methods papers from our group for extensive details about the analytical workflow [48].

#### 2.2.4. Statistical Analysis 

Significance was determined through an ANOVA test (Microsoft Excel, Redmond, CA, USA; GraphPad Prism 5.0, Prism, San Diego, CA, USA) for RBC metabolomics analyses as a function of mouse age or parabiosis. Multivariate analyses, including partial least squares discriminant analysis (PLS-DA), hierarchical clustering analyses, and heat maps, were performed with the software MetaboAnalyst 4.0 [49].

## 3. Results

### 3.1. RBCs from Aging Mice Are Characterized by Significant Proteome-Wide and Metabolic Changes in Antioxidant Systems

**Figure 1:**
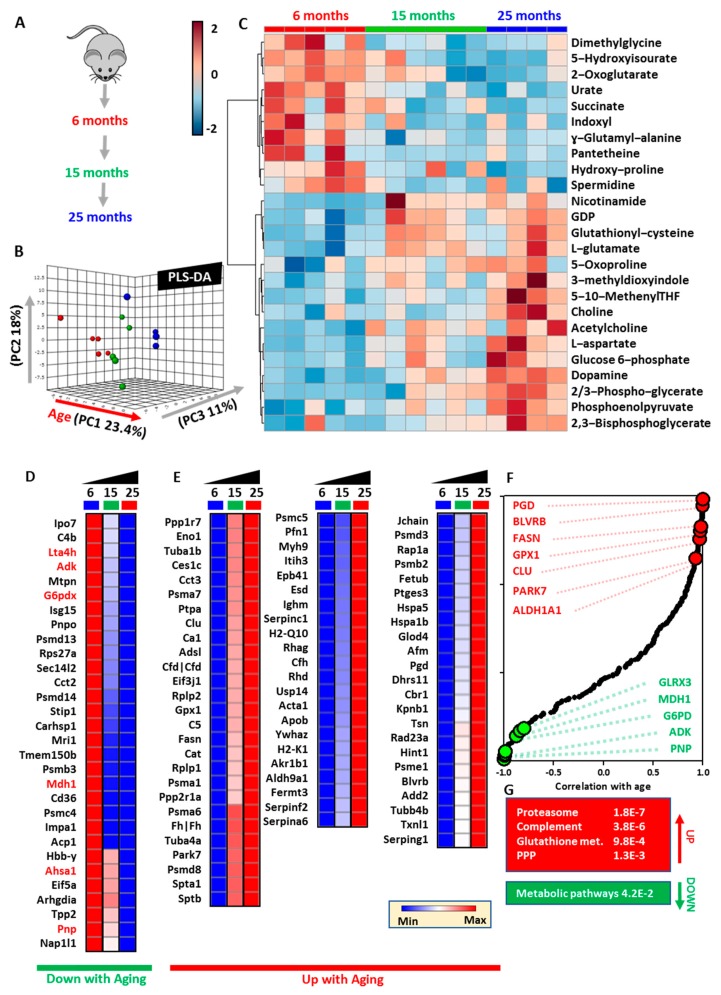
Metabolomics and proteomics of red blood cells (RBCs) from aging mice (six, 15, or 25 months old). (**A**) An overview of the experimental design. (**B**) Partial least squares discriminant analysis (PLS-DA) reveals a significant impact of mouse age on RBC metabolic phenotypes, as highlighted by the top 25 significant metabolites by ANOVA in the heat map in (**C**). Similarly, the RBC proteome was significantly impacted by the age of the animal, with a number of proteins decreasing (**D**) or increasing (**E**) with aging. Correlation analysis reveals top protein correlates (**F**) and significantly up- and downregulated pathways in aging mouse RBCs (**G**).

Metabolomics analyses were performed on RBCs from six-, 15-, or 25-month-old mice (Figure 1A). Raw data are presented in Table S1 (Supplementary Materials). Multivariate analyses revealed significant differences across samples from mice at different age groups, with aging explaining the majority of total variance (23.4% in principal component 1) in PLS-DA (Figure 1B). Hierarchical clustering analysis (Figure 1C) revealed a significant mouse age-dependent decay in RBC metabolites involved in one-carbon metabolism (e.g., dimethylglycine), carboxylic acids (2-oxoglutarate, succinate), glutathione metabolism (glutamyl-alanine, glutathione), and purine metabolism (urate, 5-hydroxyisourate). Conversely, RBCs from aged mice were characterized by increases in metabolites in the glutathione oxidation and turnover (glutathionyl-cysteine, 5-oxoproline), tryptophan and tyrosine metabolism, glycolytic metabolites (glucose 6-phosphate, phosphoglycerate isomers, phosphoenolpyruvate, 2,3-biphosphoglycerate), one-carbon and choline metabolism (methenyl-tetrahydrofolate (THF), choline, acetylcholine; Figure 1C). Proteomics analyses showed that these metabolic changes were accompanied by progressive age-related decreases in RBC levels of antioxidant enzymes (e.g., glucose 6-phosphate dehydrogenase (G6PDx)), cytosolic isoforms of Krebs cycle enzymes (malate dehydrogenase 1 (MDH1); Figure 1D). On the other hand, increases were observed in the levels of other enzymes including some involved in glycolysis (enolase (ENO1)), glutathione or NADPH-dependent antioxidant enzymes (Gpx1, catalase (Cat), aldehyde dehydrogenase 9 family member 1 (ALDH9A1), biliverdin reductase B (BLVRB), phosphogluconate dehydrogenase (PGD)), proteasome subunits (Psma1, 6, 7; Psmb2; Psmc5; Psmd3; Psme1), and heat-shock proteins (Hspa1b, Hspa5) (Figure 1E). Of note, RBCs from aging mice were characterized by increasing levels of apoptotic markers (Clusterin (Clu); Fatty Acid Synthase (Fasn), complement components (C5, Cfh) and immunoglobulin chains (Ighm, Jchain), phosphatase/kinase system (adducin (Add2); 14-3-3 protein zeta/delta - Ywhaz) and, most notably, markers of organismal aging (Park7, pregnancy zone protein (Pgzp1)). Correlation analysis between protein levels and mouse age revealed top protein correlates (F) and significantly up- and downregulated pathways in aging mouse RBCs (**G**).

### 3.2. Focus on the RBC Metabolic Pathways Impacted by Mouse Age 

#### 3.2.1. Glutathione, One-Carbon, Glycolysis, and Pentose Phosphate Pathway

**Figure 2:**
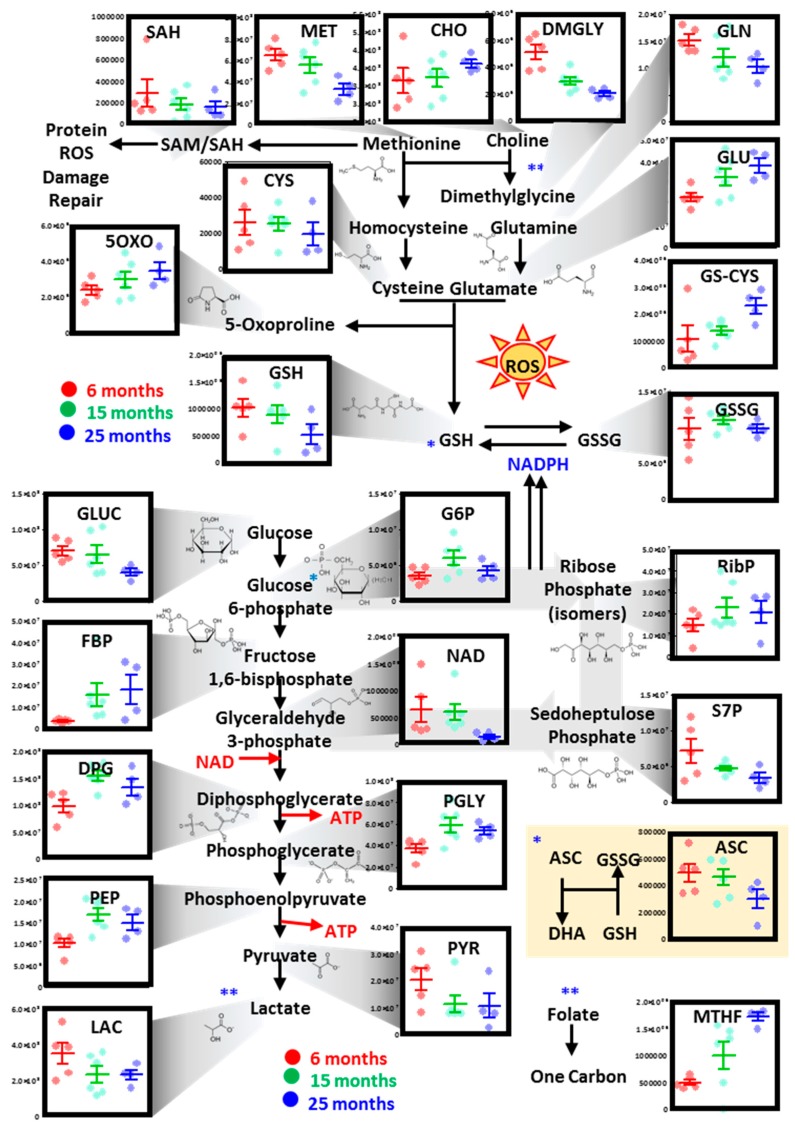
Alterations of RBC glycolysis, the pentose phosphate pathway, and glutathione and one-carbon metabolism as a function of the age of the mouse. Dots represent distinct biological replicates for six- (red), 15- (green), and 25-month-old mice (blue). * cross-talk between glutathione homeostasis and glyoxylate pathway. ** cross-talk between methionine and one-carbon metabolism.

**Figure 3:**
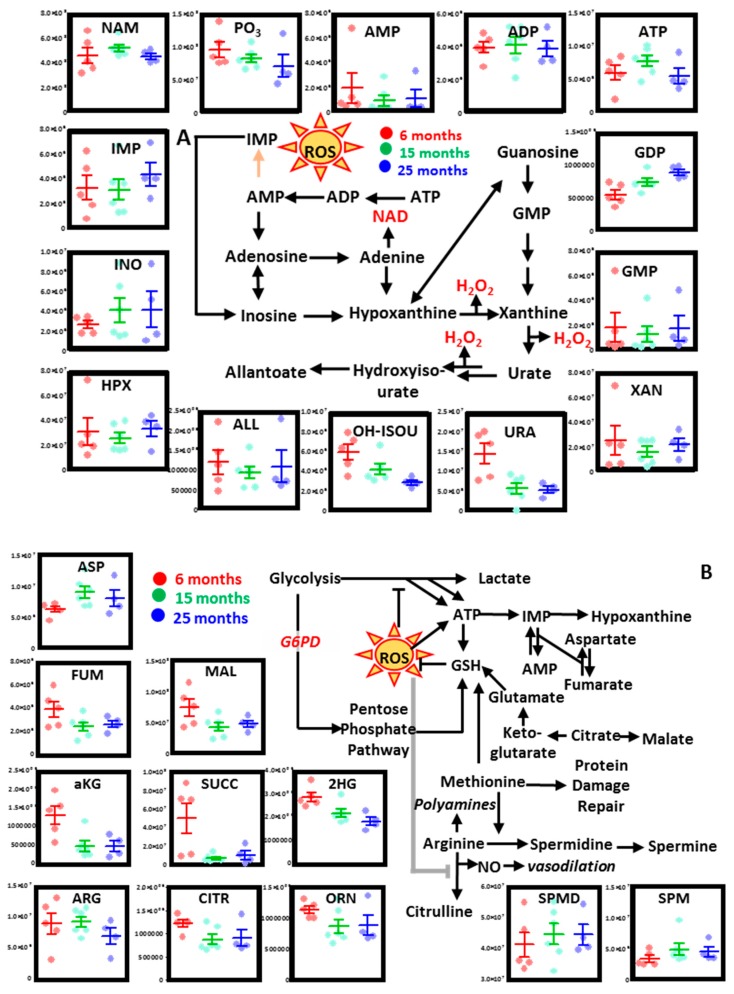
Alterations of purine metabolism (**A**), and carboxylic acid and arginine metabolism (**B**) in mouse RBCs as a function of aging. Dots represent distinct biological replicates for six- (red), 15- (green), and 25-month-old mice (blue).

**Figure 4:**
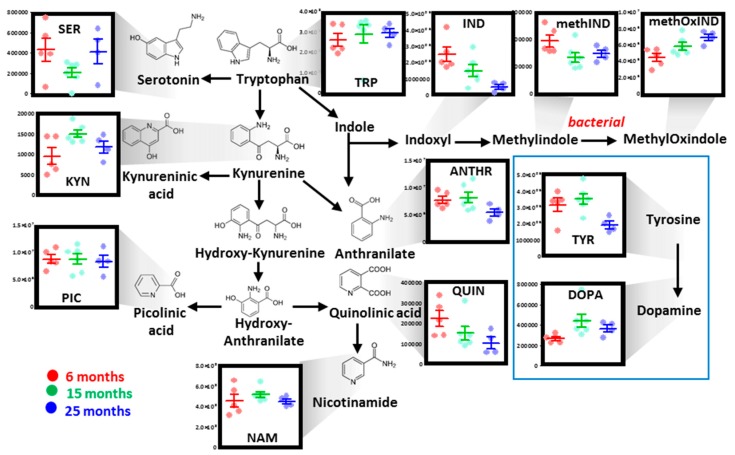
Tryptophan metabolism in mouse RBCs as a function of aging. Dots represent distinct biological replicates for six- (red), 15- (green), and 25-month-old mice (blue).

In Figure 2, Figure 3 and Figure 4, we provide an overview of the top metabolic pathways that were found to be significantly impacted by mouse age from the pathway analysis of proteomics data (Figure 1G) and multivariate analysis of metabolomics data (Figure 1B,C). Specifically, we noted that aging mouse RBCs were characterized by progressive declines of methionine, choline, and dimethylglycine (one-carbon metabolism; Figure 2). These changes were accompanied by glutamine consumption to generate glutamate, glutathione consumption as a result of increases in glutathionyl-cysteine (a marker of glutathione oxidation), and 5-oxoproline (a marker of glutathione turnover) (Figure 2). Conversely, RBC oxidized glutathione (GSSG) levels did not decline with mouse aging, resulting in GSSG/reduced glutathione (GSH) ratios increasing in older mice in comparison to young mice (Figure 2). Changes in redox homeostasis were accompanied by increased glucose consumption, as a result of (i) alterations in pentose phosphate pathway activation (higher ribose phosphate but lower sedoheptulose phosphate as a function of mouse aging), and (ii) increases in early glycolytic intermediates (hexose phosphates and triose phosphate compounds until phosphoenolpyruvate) and decreases in pyruvate and lactate (Figure 2).

#### 3.2.2. Purine Metabolism

No major changes were observed in aging mouse RBCs with respect to the total adenylate pool (ATP, ADP, AMP), although trends toward decrease were noted for AMP and ATP, especially in the oldest mice. On the other hand, significant increases in GDP, but not GMP, were observed in older mouse RBCs (Figure 3A). Purine catabolism products, including inosine monophosphate (IMP) and its breakdown and oxidation products (inosine, hypoxanthine), were higher in older mice. On the other hand, the final products of the pathway—urate, hydroxyisourate, and allantoate—were either higher in young mouse RBCs or did not change in aging mice (Figure 3A).

#### 3.2.3. Transamination, Carboxylic Acids, and Arginine Metabolism

Increases in aspartate and glutamate and decreases in alpha-ketoglutarate (aKG) are suggestive of deregulation of transamination reaction as a function of mouse age (Figure 3B). Since these reactions could be coupled to salvage of purine oxidation, it was interesting to note that carboxylates all decreased in mouse RBCs as a function of age (e.g., aKG, succinate, 2-hydroxyglutarate, fumarate, malate; Figure 3B). Finally, both 15- and 25-month-old mice were characterized by deregulation of arginine metabolism, which resulted in lower levels of citrulline and ornithine, but higher levels of spermidine and spermine in RBCs from old mice (Figure 3B).

#### 3.2.4. Tryptophan, Tyrosine, and Indole Metabolism

Multivariate analysis highlighted significant age-related changes in the levels of RBC metabolites of tryptophan and indoles, with kynurenic acid and quinolinic acid increasing and decreasing, respectively as a function of mouse age (Figure 4). Indole metabolites—likely products of bacterial origin—were found to decrease and increase in aged mouse RBCs (Figure 4). Finally, tyrosine decreases in 25-month-old mice were accompanied by significant increases in the levels of its byproduct dopamine (Figure 4).

#### 3.2.5. Parabiosis Only Partially Restores Metabolic and Proteome-Wide Defects in RBCs from Aging Mice

**Figure 5:**
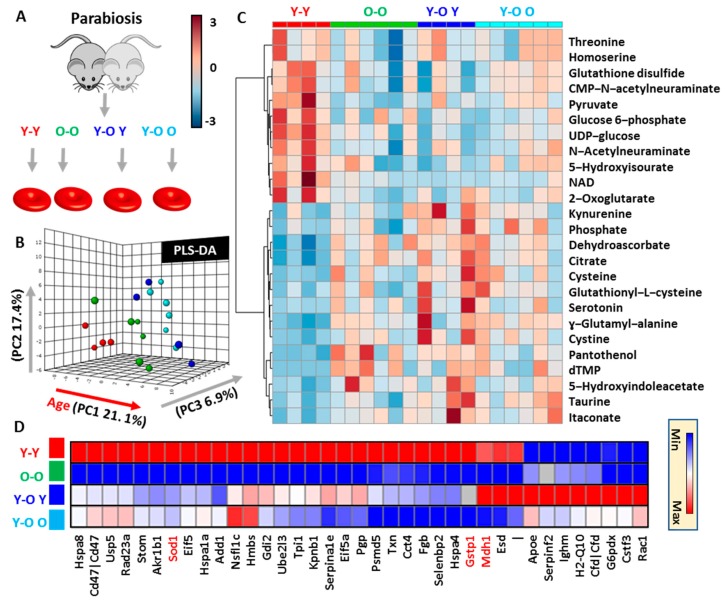
Metabolomics and proteomics analyses of RBCs in isochronic and heterochronic parabiotic mice. In (**A**), an overview of the experimental design. In (**B**), partial least square-discriminant analysis (PLS-DA) from the metabolomics data. In (**C**,**D**), top significant metabolic and protein changes in the parabiosis experiments are presented in the form of a heat map.

After appreciating proteomics and metabolic changes in RBCs from aging mice, we questioned whether some of these age-related phenotypic changes could be reversed by physically connecting the circulatory systems of young and old mice—a practice referred to as parabiosis [36]. In the present study, the circulatory systems of two young or old mice (isochronic Y–Y or O–O, respectively) or one young and one old mouse (heterochronic Y–O) were surgically connected (Figure 5A), prior to blood collection from either mouse and subsequent metabolomics (Supplementary Table S2) and proteomics (Supplementary Table S3) analyses. In heterochronic mice, RBCs were either drawn from the young (Y–O Y) or the old (Y–O O) mouse. Once again, metabolomics data clearly indicated an impact of mouse age across principal component 1 (PC1; 21.1% of the total variance). On the other hand, PC2 and PC3 accounted for 17.4% and 6.9% of the total variance, respectively, mostly as a result of biological variability across mice (PC1) and the effect of parabiosis (PC3) (Figure 5B). Hierarchical clustering of the top 25 metabolites by ANOVA is shown in Figure 5C. Results confirmed a significant impact of aging on glutathione homeostasis, glycolysis, and the pentose phosphate pathway. Only a subgroup of age-related changes were reversed in heterochronic mice, when compared to isochronic old–old mice. Similarly, the proteomes of RBCs from heterochronic mice (young–old mice) were characterized by higher levels of antioxidant enzymes (superoxide dismutase 1 - Sod1) and Cd47, a “do not eat me signal” that prevents the untimely removal of the RBC from the bloodstream via phagocytosis (Figure 5D). Parabiosis also resulted in increased levels of G6PD in the RBC from young–old mice, especially in Y–O Y mice (Figure 5D). Heterochronic mice (both Y–O O and Y–O Y) had young levels of hydroxymethylbilane synthase (HMBS), an enzyme critical in heme metabolism whose mutation is associated with acute porphyrias (Figure 5D). Similarly, heterochronic parabionts had normal levels of NSFL1 cofactor p47 (NSFL1C), a competitive inhibitor of cathepsin proteases [50].

#### 3.2.6. Parabiosis Only Partial Rescues the Age-Dependent Changes in RBC Metabolism

**Figure 6:**
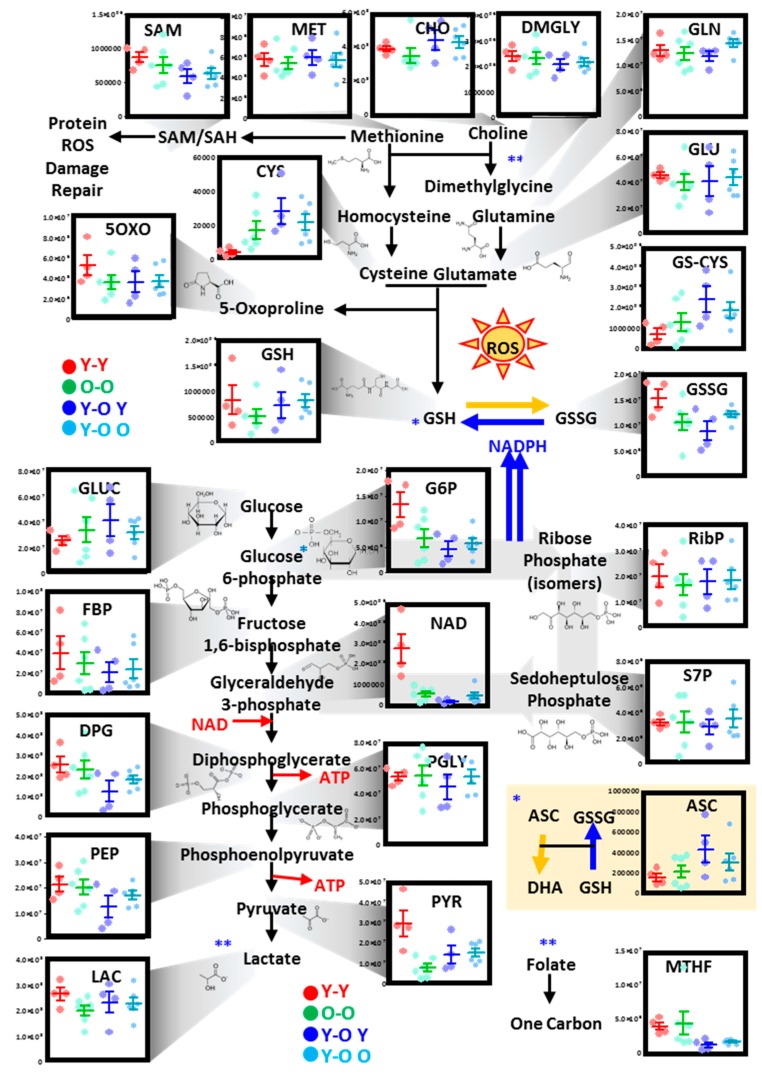
Alterations of RBC glycolysis, the pentose phosphate pathway, and glutathione and one-carbon metabolism in isochronic and heterochronic parabiotic mice. Dots represent distinct biological replicates for isochronic young (Y)–young (red) or old (O)–old (green), or heterochronic Y–O mice with blood being drawn from the young mouse (blue) or the old mouse (light blue). * cross-talk between glutathione homeostasis and glyoxylate pathway. ** cross-talk between methionine and one-carbon metabolism.

Parabiosis partially corrected the metabolic defect in one-carbon metabolism, by normalizing the levels of methionine and increasing RBC levels of choline (Figure 6). However, age-dependent decreases in *S*-adenosylmethionine (SAM) and dimethylglycine were aggravated in Y–O mice (Figure 6). Similar increases were observed in Y–O mice with respect to cysteine and glutathionyl-cysteine, despite an apparent normalization of the levels of reduced, but not oxidized glutathione in comparison to O–O mice (Figure 6). Levels of 5-oxoproline were decreased, while ribose phosphate increased in O–O and Y–O mice (Figure 6), a trend inconsistent with that observed in RBCs from aging mice (Figure 2), likely as a confounding effect of the surgical practice of parabiosis. On the other hand, parabiosis decreased the levels of early glycolytic metabolites and partially normalized the levels of late glycolytic products pyruvate and lactate back to young mouse RBC levels (Figure 6). Perhaps the most striking finding in parabiotic mice was that RBC levels of NAD dropped significantly in old mouse RBCs and were not improved by parabiosis.

**Figure 7:**
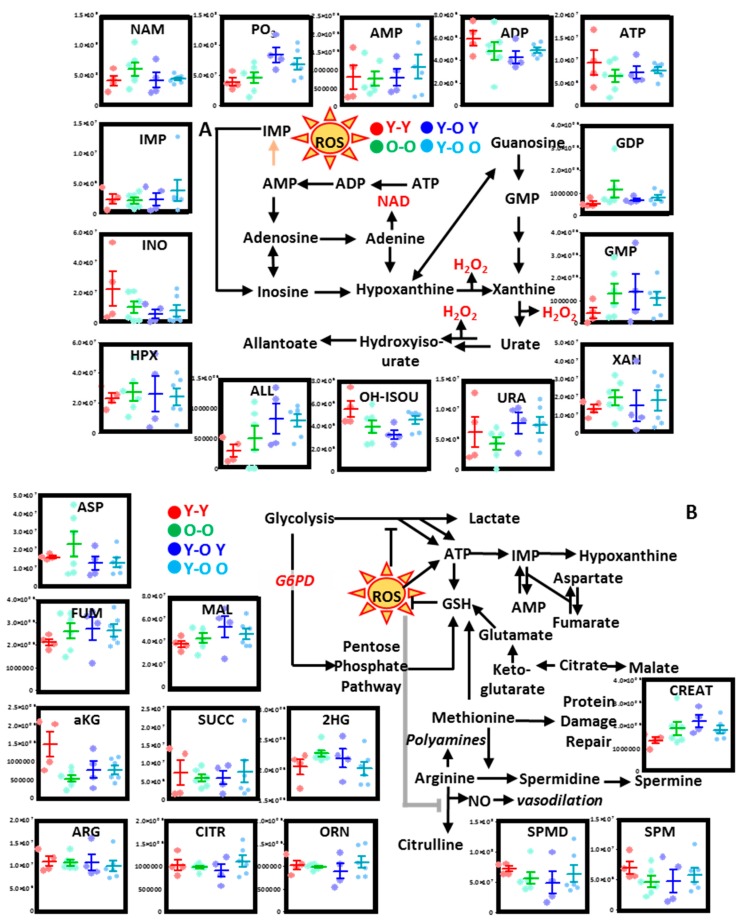
Alterations of RBC purine metabolism (**A**), and carboxylic acid and arginine metabolism (**B**) in isochronic and heterochronic parabiotic mice. Dots represent distinct biological replicates for isochronic young–young (red) or old–old (green), or heterochronic Y–O mice with blood being drawn from the young mouse (blue) or the old mouse (light blue).

**Figure 8:**
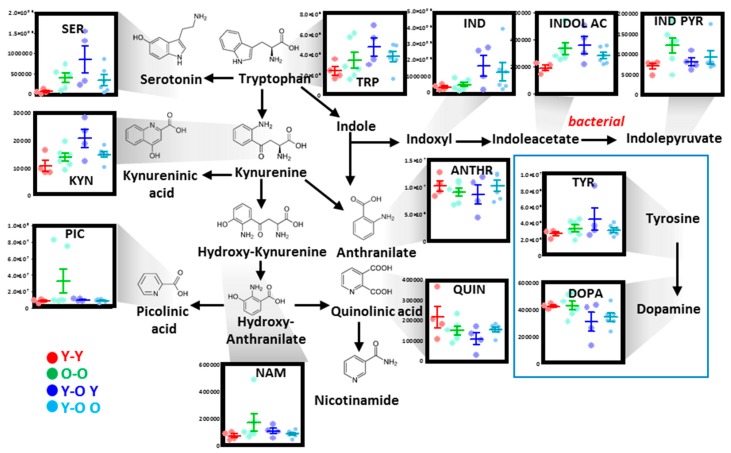
Alterations of RBC tryptophan metabolism in isochronic and heterochronic parabiotic mice. Dots represent distinct biological replicates for isochronic young–young (red) or old–old (green), or heterochronic Y–O mice with blood being drawn from the young mouse (blue) or the old mouse (light blue).

Age-related changes in parabiotic mouse RBC purines mostly confirmed and in part expanded the observation from aging mice (Figure 7A). In particular, in this set of experiments, RBCs from O–O parabiotic mice had lower levels of high-energy phosphate purines (ATP and ADP), a trend that was not reversed by parabiosis in heterochronic mice. On the other hand, parabiosis seemed to aggravate age-associated purine oxidation from urate/5-hydroxyisourate to allantoin (Figure 7A). Similar considerations can be made for the RBC levels of carboxylic acids (especially fumarate, malate, and aKG), polyamines, and creatine (arginine metabolite) (Figure 7B). Finally, RBC levels of tryptophan and indole metabolites increased in O–O compared to Y–Y mice, a trend that was further exacerbated in heterochronic mice, with the exception of the neurotoxic picolinic acid and NAD-precursor nicotinamide (Figure 8).

## 4. Discussion

In the present study, we analyzed the metabolome and proteome of RBCs from aging mice. Expectedly, we found that RBC antioxidant capacity declined as a function of mouse age, a phenomenon that was in part warranted by progressive declines in glutathione pools, in part by deregulated PPP at the protein (G6PD) and metabolic level. Prior work from the late 1970s/early 1980s showed age-dependent declines in G6PD activity [20,22] and glutathione pools [19,20,21]. The results are consistent with classic literature and recent observations in RBCs from Down syndrome [30] and non-trisomic subjects [29]. Expanding on the literature, we report that dysregulation of glutathione metabolism was in part explained by increases in oxidation and turnover of glutathione, with 5-oxoproline representing a metabolic bottleneck in RBCs owing to the lack of a functional oxoprolinase [51]. Increased glutaminolysis and glutamate/aspartate levels in the face of higher levels of cysteine were detected in the erythrocytes of aging mice, a phenotype consistent with decreased glutathione synthesis [52,53] and dysregulated transamination (also manifesting in low levels of aKG in RBCs from 15- and 25-month-old mice). Since glutathione synthesis is an ATP-dependent process, it was interesting to note that the total ATP pool was decreased in RBCs from isochronic O–O parabiotic mice, with trending decreases of ATP and AMP in RBCs from aging, non-parabiotic mice. Decreases in late glycolysis were noted despite an apparent rerouting of glucose moieties toward glycolysis, as apparent by the accumulation of early glycolytic intermediates. A tentative explanation is due to potential decreases in glucose uptake (as intracellular glucose levels declined as a function of mouse age), since the glucose transporter (Solute Carrier Family 2 Member 1- SLC2A1) was reported to be controlled transcriptionally (at earlier erythropoietic stages) by redox sensing transcriptional regulators such as ataxia–telangiencatasia mutated (ATM) [54], which also regulates G6PD at the transcriptional level [55]. On the other hand, it was shown in other systems that late glycolytic enzymes such as pyruvate kinase—where a metabolic bottleneck was observed in RBCs from old mice in the present study—can be regulated via the mechanism of redox-regulated glutathionylation [56]. However, in the present study, no significant age-dependent increase in pyruvate kinase glutathionylation was observed in old mouse RBCs, which were instead characterized by progressive oxidation and higher levels of glutathionylation of cysteine 94 of hemoglobin beta (data not shown)—one of the main redox-sensitive cysteines in RBCs [57,58]. An alternative explanation of this apparent blockade in late glycolysis is that other redox-sensitive thiols (e.g., Cys152 and 156 of glyceraldehyde 3-phosphate dehydrogenase [59]) could be oxidized in RBCs as a function of aging, although proteomics data did not confirm that in this study.

Alternatively, mouse aging may result in the depletion of cofactors that are essential to sustain glycolysis, such as nicotinamide adenine dinucleotide (NAD). Notably, NAD levels decreased with mouse age and were the lowest in 25-month-old mice, a phenotype that was not corrected by parabiosis. This is interesting owing to the increasingly appreciated link between aging and NAD depletion [60,61,62,63], an observation that fueled the industry of supplements based on NAD precursors. Even more intriguingly, NAD is a downstream product of tryptophan metabolism, a pathway that is significantly impacted by aging and inflammation [64,65]. In Down syndrome, increased tryptophan oxidation as a function of interferon-induced indole 2,3-dioxygenase (IDO1) activity results in the accumulation of neurotoxic metabolites like kynurenine and picolinic acid [66], potential contributors to the neurocognitive decline observed in the Down syndrome population [30,35] and, in general, during aging. While parabiosis had arguably little to no effect on most metabolic pathways, it is worthwhile to note that RBC levels of picolinic and quinolinic acid, but not kynurenic acid, were normalized in heterochronic parabiotic mice. Of note, the tryptophan/kynurenine axis was recently associated with a relay axis that regulates arginine catabolism to polyamines as a function of IDO1 and arginase 1 activity in dendritic cells [34], an observation that is herein phenocopied in RBCs from aging mice and only partially normalized by parabiosis.

Recent studies highlighted the potential role as signaling molecules of tryptophan-derived indoles, metabolites that can be generated by some Gram-negative bacteria in the gut microbiome [64,67,68]. Although merely speculative at this stage, it is interesting to note that indole metabolites were significantly impacted by the age of the animal in RBCs, which also circulate in microcapillaries in the gut and are, thus, indirectly exposed to bacterial metabolites. As such, the observed changes in metabolites of potential bacterial origin in aged mouse RBCs could represent a previously unappreciated marker of gut dysbiosis in “inflammaging” that warrants further investigation in the future.

Increases in circulating levels of pro-inflammatory cytokines such as interleukin-1 (IL-1) and -18 were proposed as mediators of the aging process [69]. Increases in circulating levels of IL-1β were shown to skew hematopoiesis toward the myeloid lineage by mechanisms involving the activation of a PU.1-dependent gene program [17]. This mechanism could potentially explain the phenomenon of “anemia in the elderly” [70], a condition that is found in ~80% of elderly patients and at least in part explained by progressive iron, folate, and vitamin B12 deficiency [18]. Of note, both folate and vitamin B12 are essential players in one-carbon metabolism, a pathway that fuels the synthesis of purines and *S*-adenosylmethionine for protein, DNA, and RNA methylation purposes. Indeed, dietary manipulation of folates results in altered hematopoiesis [71], and dysregulation of this pathway owing to dosage increase of cystathionine beta synthase in Down syndrome results in macrocytic anemia and homocystinuria [72]. Notably, in the present study, one-carbon metabolism (including critical players methionine, choline, dimethylglycine, and methenyl-THF) was the most dysregulated pathway in the aged mouse RBC. This is particularly relevant in RBCs, since increased methionine consumption is critical to the RBC capacity to repair isoaspartyl damage to proteins as a function of oxidant stress by mechanisms involving the protein l-isoaspartyl methyltransferase (PIMT1) [33,40]. Indeed, genetic ablation of PIMT1 results in 100% mortality by 6–8 weeks of age because of seizures following unsustainable oxidant damage to the central nervous system [73,74,75,76]. In this view, it is interesting to note that (i) parabiosis was previously shown to correct, at least in part, the neurocognitive defect in the aging mouse [37], and that (ii) in the present study, parabiosis effectively replenished the RBC levels of methyl-group donors methionine and choline, although it was insufficient to fully normalize one-carbon metabolism in the heterochronic mouse RBC. Overall, in the context of the literature and the data presented herein, further studies investigating the potential linkage between RBC one-carbon metabolism, oxidant stress, and protein damage repair in aging seem appropriate.

Anemia in the elderly is associated with a compromised capacity to sustain sufficient tissue hypoxia. Of note, RBC levels of carboxylic acids were previously associated with high-altitude [77] or hemorrhagic hypoxia [78], while circulating levels of carboxylic acids are indicators of pathological mitochondrial dysfunction in aging, trauma, and inflammation [79,80,81,82]. In this study, RBC levels of carboxylic acids were significantly decreased as a function of mouse age. However, a limitation of our analysis and its interpretation is that plasma samples were not available to determine matching levels of these metabolites. Still, the appreciation of significantly lower levels of cytosolic isoforms of Krebs cycle enzymes such as MDH1 is consistent with altered NAD/NADP-dependent metabolism [83] in the RBC from the aged mouse. In the mature RBC, these alterations manifest themselves as a result of the dysregulation of redox-regulated pathways related to purine oxidation and salvage [44], a phenomenon that was observed in the RBC of the aged mouse, which negatively impacted the total adenylate pool and, thus, the energy status of the cell, independently of parabiosis.

RBCs from old mice were characterized by higher levels of proteasome subunits and cellular senescence signaling components (clusterin [84]), suggestive of potential compensatory mechanisms to cope with increased oxidant stress in the face of decreased G6PD levels and PPP activation.

Recently, debate sparked over the opportunity to “rejuvenate” old subjects by transfusion of blood from young donors [85,86,87]. Commercial companies jumped on the opportunity to leverage a well-established medical practice to profit on a potentially game-changing therapy for aging. However, recent critical reassessment of currently available literature by the Food and Drug Administration “cautions consumers against receiving young donor plasma infusions that are promoted as unproven treatment for varying conditions” at this stage [88]. In this context, it is worthwhile to note that the present study did not focus on the potential impact of transfusion of blood from young mice into old mice; rather, it tested the impact of heterochronic parabiosis in the context of aging. As such, caveats should be acknowledged such as the likely biological impact of the parabiosis procedure per se on mouse RBCs. Finally, it should be noted that transfusion of young mouse RBCs into old mice would represent an “acute” procedure, while parabiotic mice were surgically connected for ~2 months. In this view, making our report represents an assessment of the impact of a “chronic” procedure/phenotype; thus, any parallelism to the potential impact of transfusion of young blood into old subjects would be merely speculative.

## 5. Conclusions

In the present study, we report the results from metabolomics and proteomics analyses of RBCs from six-, 15-, and 25-month-old mice. As a result, we identified a significant decline in the glutathione and NADPH-dependent antioxidant capacity of the RBCs from aged mice. This phenomenon was accompanied by declines in one-carbon metabolism, and increased purine and tryptophan oxidation, with the latter being associated with significant decreases in the NAD pool. Parabiosis experiments were performed to investigate whether circulation in the bloodstream of a younger mouse was sufficient to restore the metabolic phenotype of an RBC from an old mouse into a younger phenotype. However, parabiosis only partially rescued the alterations in one-carbon metabolism, although it had minimal to no impact on glutathione homeostasis, the PPP, and oxidation of purines and tryptophan, which were instead aggravated in heterochronic parabiotic mice. Compensatory mechanisms seemed to emerge in the RBC from aged mice, such as increased utilization of one-carbon compounds from methyl-group donors, and increased levels of proteasomal and apoptotic cascade components in the mature RBC from aged mice when compared to their counterparts from younger mice. Future studies will be necessary to mechanistically expand on the present observations, such as investigations aimed at defining (i) the role of G6PD activity (or lack thereof) in the mature RBC as a function of aging, (ii) the role of these potential compensatory mechanisms involving the use of one-carbon moieties, proteasome activation, or cellular senescence cascades in the aging organism, and, most importantly, (iii) whether and to what extent these critical players of RBC redox and energy homeostasis contribute to aging-associated comorbidities.

## Supplementary Materials

The following are available online at https://www.mdpi.com/2072-6643/11/6/1337/s1: Table S1: Metabolomics report for aging mouse RBCs; Table S2: Metabolomics report for parabiotic mouse RBCs; Table S3: Proteomics report for aging and parabiotic mouse RBCs.
